# Items

**Published:** 1878-07

**Authors:** 


### Items.

HOMŒOPATHIC "SOUL MEDICINE."

"In allopathy the soul is nowhere; in homœopathy the state of the soul or mind is a *sine quâ non.*

"Allopathy has no means of affecting the soul or mind, except those of a moral kind; whereas homœopathic medicines act upon the spirit or soul of man, and through it and by means of it, and with a certainty which is as remarkable as it is true.

"By way of illustrating the power of homœopathic medicines over the mind and its affections, I shall give the following example: A favorite cat of my own had kittens; all were drowned but two; then one was given away, and ultimately the remaining one was given to a friend. The mother of the kittens became *inconsolable*, and went all over the house, mourning her loss in unmistakable *tones of grief*, for five days and nights, making night hideous with her cries. One globule of *Ignatia* cured her in half an hour, as she never cried again."—(Skinner's "Diseases of Women," p. 27; Porter & Coates, Phila. *Phil. Med. Times*, June 8, 1878.)

THE following named physicians constitute the newly appointed Medical Board of the Cook County Hospital: Drs. McWilliams, Lee, Jacobson, Hessert, Guerin, Baxter, Fenger, Ross, Gunn, Parkes, Quine, Bevan and Isham.

IF the end of the uterus appears as hard as the end of your nose, pregnancy should exist. If it appears as soft as your lips, the uterus probably contains a fœtus.—W. GOODELL.

